# The Vibrational Signature of Alzheimer’s Disease: A Computational Approach Based on Sonification, Laser Projection, and Computer Vision Analysis

**DOI:** 10.3390/biomimetics10120792

**Published:** 2025-11-21

**Authors:** Rubén Pérez-Elvira, Javier Oltra-Cucarella, María Agudo Juan, Luis Polo-Ferrero, Raúl Juárez-Vela, Jorge Bosch-Bayard, Manuel Quintana Díaz, Jorge de la Cruz, Alfonso Salgado Ruíz

**Affiliations:** 1 Department of Psychobiology, Faculty of Psychology, Pontifical University of Salamanca, 37002 Salamanca, Spain; asalgadoru@upsa.es; 2 Neuropsychophysiology Laboratory, NEPSA Rehabilitación Neurológica, 37003 Salamanca, Spain; mjagudojuan@gmail.com; 3 Department of Health Psychology, Miguel Hernández University, 03202 Elche, Spain; joltra@umh.es; 4 Department of Nursing and Physiotherapy, University of Salamanca, 37007 Salamanca, Spain; pfluis@usal.es; 5 Department of Nursing, Faculty of Health Sciences, University of La Rioja, 26004 Logroño, Spain; raul.juarez@unirioja.es; 6 Faculty of Psychology, University of Oldenburg, 26111 Oldenburg, Germany; oldgandalf@gmail.com; 7 Intensive Care Unit, La Paz University Hospital, 28046 Madrid, Spain; mquintanadiaz@gmail.com; 8 Huanjiang Laboratory, Center of Research on Microgrids (CROM), Zhuji 311800, China; jorge.delacruz@ieee.org

**Keywords:** Alzheimer’s disease, sonification, electroencephalogram, laser projection, biomarkers

## Abstract

Alzheimer’s disease (AD) is the most prevalent form of dementia, and accessible biomarkers for early detection remain limited. This study introduces a biomimetic approach in which brain electrical activity is transformed into sound and vibration, emulating natural sensory encoding mechanisms. Resting-state EEG recordings from 36 AD patients and 29 healthy controls were averaged by group, directly sonified, and used to drive a membrane–laser system that projected dynamic vibrational patterns. This transformation mirrors how biological systems convert electrical signals into sensory representations, offering a novel bridge between neural dynamics and physical patterns. The resulting videos were processed through adaptive binarization, morphological filtering, and contour-based masking. Quantitative descriptors such as active area, spatial entropy, fractal dimension, and centroid dynamics were extracted, capturing group-specific differences. A Random Forest classifier trained on these features achieved an accuracy of 0.85 and an AUC of 0.93 in distinguishing AD from controls. These findings suggest that EEG sonification combined with vibrational projection provides not only a novel non-invasive biomarker candidate but also a biomimetic framework inspired by the brain’s own capacity to encode and represent complex signals.

## 1. Introduction

Alzheimer’s disease (AD) represents the most common cause of dementia worldwide, affecting millions of people and constituting a significant challenge for public health systems [1]. Early and accurate detection of AD is crucial to implement interventions that can delay its progression and improve patients’ quality of life. In this context, the identification of non-invasive biomarkers has become a priority in neuroscientific research [2].

Traditionally, the diagnosis of AD has been based on clinical assessment [3] and neuropsychological testing [4,5,6], complemented by neuroimaging techniques such as magnetic resonance imaging and positron emission tomography [7,8]. However, these techniques can be expensive, invasive and not always accessible. Therefore, the search for peripheral and non-invasive biomarkers that allow earlier and more accessible detection of the disease has intensified [9].

Among the emerging biomarkers, electroencephalography (EEG) has gained attention due to its ability to reflect brain electrical activity in real time, its low cost, and its non-invasive nature [10,11,12]. Recent studies have shown that certain EEG characteristics, such as decreased activity in fast frequency bands and increased activity in slow bands, are associated with AD progression [13,14]. In addition, more advanced EEG analyses, such as assessment of signal complexity [15,16] and functional connectivity [17], have shown potential to differentiate between healthy individuals and those with mild cognitive impairment or AD [18]. The utility of EEG in the search for biomarkers is enhanced by its ability to detect neurophysiological changes even in preclinical stages of the disease [19]. For example, it has been observed that alterations in the timing and complexity of EEG signals may precede the manifestation of clinical symptoms, suggesting their potential for the early detection of AD [15,20]. In this context, EEG offers a promising tool for the identification of non-invasive biomarkers in AD, providing valuable information on brain dynamics and allowing accessible and early assessment of the disease.

While this is true, conventional EEG analyses have some limitations that could reduce their clinical usefulness when diagnosing pathologies such as AD. Conventional spectral analysis fragments signals into discrete time windows to extract their characteristics, which could lead to the loss of information about global dynamics and long-range temporal correlations that characterize brain activity [21]. Furthermore, statistical measures extracted from two-dimensional signals may not fully capture the inherent complexity of brain activity patterns or may ignore some relevant dynamics [22,23]. These limitations call for innovative analysis approaches that preserve the complete temporal structure of brain signals and, if present, reveal hidden neurophysiological information. Combined modalities such as functional Near-Infrared Spectroscopy–EEG systems have been proposed to overcome some of these limitations, offering complementary spatial and hemodynamic information [24].

Sonification, defined as the representation of information through sound, has emerged as a promising technique for the analysis of complex signals in neuroscience [25,26]. When applied to the analysis of brain electrical activity, this technique capitalizes on the human auditory system’s capacity to detect subtle temporal patterns and dynamic relationships that may remain unnoticed in conventional visual representations [27]. In the context of EEG signals, sonification enables the transformation of brain electrical activity into audible representations that preserve the original temporal characteristics of neural patterns.

Initial methods include spectral mapping sonification, which converts specific frequency components into audible tones, and distance mapping sonification, which encodes spatial relationships between electrodes into distinguishable sound patterns [25]. More specialized applications have employed auditory alarms for surgical instruments, model-based systems for seizure analysis, and discrete frequency transformations for brain–computer interface development [28]. An innovative approach is bump sonification, introduced by Vialatte et al. [26], which employs sparse time–frequency representations of EEG via “bump” modeling to generate musically structured sequences with clinical interpretability. This approach has demonstrated discriminative power between patients with mild cognitive impairment and healthy controls, yielding statistically significant differences in sample entropy, number of sound events, and synchronization measures, with a classification accuracy of 89% in human perception tests.

In parallel, direct sonification methods, in which EEG signal amplitudes are linearly mapped to sound pressure levels, have proven particularly valuable for maintaining the temporal fidelity of brain activity [29,30]. Taken together, these findings suggest that sonification represents a viable tool for the detection of subtle neurophysiological patterns that may not be evident in conventional visual analyses.

The aim of this work is to investigate whether laser-projected vibrational patterns derived from average EEG sonification of groups of subjects contain quantifiable differences capable of discriminating between healthy controls and patients with Alzheimer’s disease. Through a computational approach combining image processing, spatial analysis and automatic classification, we seek to lay the foundation for the future development of dynamic, visual and non-invasive biomarkers of brain function. This work represents a proof-of-concept study aimed at establishing the basic feasibility of a novel biomimetic methodology before proceeding to individual-level validation.

## 2. Materials and Methods

### 2.1. Subjects

This study was based on resting-state EEG data in the eyes-closed condition, obtained from the OpenNeuro ds004504 public dataset, version 1.0.7 [31]. This dataset comprises EEG recordings from a total of 88 subjects, of which 65 were selected for the present analysis: 36 patients diagnosed with AD and 29 healthy control subjects (NC), matched for age. The EEG recordings were acquired at the 2nd Department of Neurology of AHEPA University Hospital (Thessaloniki, Greece) using a Nihon Kohden EEG-2100 clinical system with 19 scalp electrodes positioned according to the international 10–20 system and two mastoid references (A1–A2). Data were collected at a sampling rate of 500 Hz with 10 μV/mm sensitivity, a time constant of 0.3 s, and a high-frequency filter of 70 Hz. Each session lasted approximately 12–14 min, during which participants remained seated with eyes closed.

The raw data were converted to BIDS-compliant .set files, and a preprocessed version is also available in the derivatives folder of the dataset. The preprocessing pipeline included a 0.5–45 Hz Butterworth band-pass filter, re-referencing to A1–A2, Artifact Subspace Reconstruction (ASR) for the removal of high-variance segments, and Independent Component Analysis (ICA) using the RunICA algorithm. Artifactual components (e.g., eye and jaw movements) were automatically detected and rejected via the ICLabel classifier implemented in EEGLAB.

Full methodological details and validation of this dataset are described in the Data Descriptor article “A Dataset of Scalp EEG Recordings of Alzheimer’s Disease, Frontotemporal Dementia and Healthy Subjects from Routine EEG” [31].

#### Features of the Participants

AD group: 36 patients diagnosed with AD, with a mean age of 66.4 years (SD = 7.9) and a mean Mini-Mental State Examination (MMSE) score of 17.75 (SD = 4.5). The median duration of illness in the AD group was 25 months (interquartile range: 24–28.5 months).

Group NC: 29 healthy subjects, with a mean age of 67.9 years (SD = 5.4) and an MMSE score of 30.

### 2.2. Procedure

The conceptual basis of our approach is founded on the analogy between neural coding and sensory transduction mechanisms. In biological systems, oscillatory neural activity encodes information through frequency, amplitude, and phase modulation, processes that underlie perception and cognition [32]. By directly mapping EEG dynamics to the acoustic and vibratory domains, the sonification step preserves the temporal and spectral characteristics of neural signals, allowing their physical representation as patterns perceptible to the senses. This transformation does not aim to replicate neural computation itself, but rather to emulate the way sensory systems translate distributed electrical activity into structured and perceptible representations. In the context of AD, in which neural synchronization and oscillatory coherence are altered [33], this translation would provide a means to visualize alterations in large-scale network dynamics.

**Figure 1:**
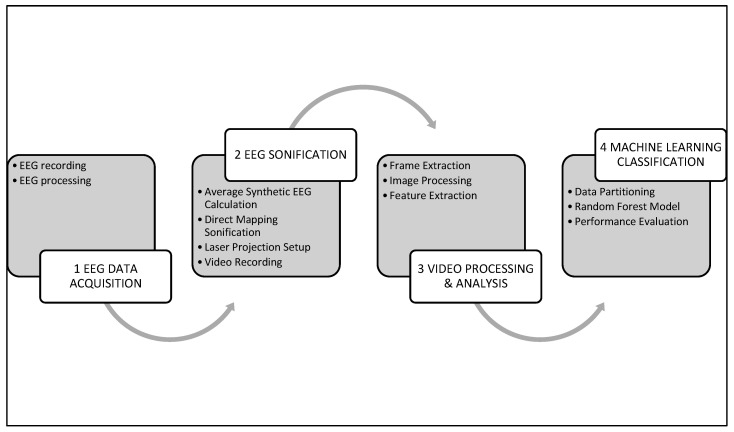
Procedure flowchart.

Figure 1 shows a flowchart of the entire procedure used in this study.

#### 2.2.1. EEG Recording Procedures

EEG recordings, as mentioned in the database reference [31], were performed at the 2nd Department of Neurology, AHEPA University Hospital, Thessaloniki, Greece, using a Nihon Kohden 2100 clinical EEG system. A monopolar setup was used with 19 scalp electrodes placed according to the international 10–20 system (Fp1, Fp2, F7, F3, Fz, F4, F8, T3, C3, Cz, C4, T4, T5, P3, Pz, P4, T6, O1 and O2), plus two reference electrodes on the mastoids (A1 and A2). The sampling rate was 500 Hz, with a resolution of 10 µV/mm. Each recording had an average duration of 13.5 min for the EA group (range: 5.1–21.3 min) and 13.8 min for the NC group (range: 12.5–16.5 min).

#### 2.2.2. EEG Processing

All EEG recordings used in the present study were subjected to standardized processing in order to optimize signal quality and minimize the influence of artifacts. Initially, the EEG signals were traced back to the average reference montage, which reduces the dependence on the reference point and provides a more balanced estimation of the brain electric field, favoring the analysis of global patterns of brain connectivity and oscillation [34].

Subsequently, for the cleaning of artifacts, the Artifact Subspace Reconstruction (ASR) method was applied. ASR is an automated technique that identifies periods of anomalous activity in the EEG based on the deviation of the statistical characteristics of the signal and corrects them by reconstructing the affected portions from uncontaminated component subspaces. This approach allows preserving most of the genuine brain signal while eliminating large amplitude artifacts that could bias the subsequent analysis [35].

#### 2.2.3. EEG Sonification

To physically represent the dynamic differences between healthy subjects and patients with Alzheimer’s disease, a sonification process was applied to average EEG recordings of each group.

##### Average Synthetic EEG Calculation

First, EEG recordings in EDF format were obtained from each subject. Subsequently, the data from all subjects in the NC group were averaged channel by channel. The same procedure was applied to the subjects in the AD group. Averaging was performed in the time domain, without applying any specific band-pass filtering, thereby preserving the maximum amount of information across the frequency spectrum. This procedure yielded a synthetic EEG signal representative of the overall group dynamics for each condition.

##### Conversion of EEG Signal to Sound File

The synthetic EEG signals corresponding to each group were converted into audio files through a direct sonification process. We applied Direct Mapping Sonification [25,27,29], a process widely used to preserve the original temporal morphology of biological signals [27]. The amplitude of the EEG time series was mapped linearly to sound pressure levels, without applying frequency modulation or additional signal transformations. Before final rendering, the amplitude values were normalized to prevent signal saturation and clipping, ensuring that all data points remained within the dynamic range suitable for audio reproduction.

The sonification was implemented in Python (version 3.13) using open-source scientific libraries, including MNE-Python for EEG handling, NumPy and SciPy for signal processing and waveform generation, and the scipy.io.wavfile module for audio rendering in .WAV format. A lightweight graphical interface for selecting EEG directories and processing parameters was developed using Tkinter (version 8.7). The audio files were generated at a 44.1 kHz sampling rate to preserve temporal resolution, normalized to prevent clipping, and saved as 16-bit PCM signals to ensure compatibility with standard audio processing hardware.

Each synthetic EEG signal was thus rendered into an audible waveform, maintaining the original temporal structure of the brain activity patterns while allowing subsequent physical projection through vibrational media. For this purpose, they were converted into audio files (.WAV; Alzheimer’s: File S1, Controls: File S2), one for each group.

##### Vibration-Modulated Laser Projection and Visual Capture

**Figure 2:**
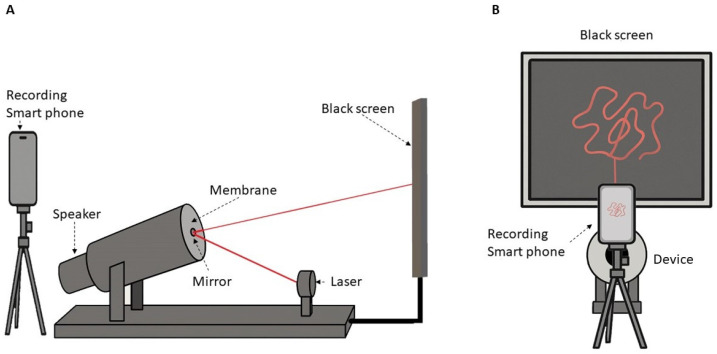
Schematic view of the experimental setup. (**A**) Schematic lateral view of the experimental setup, illustrating all components of the device, including the speaker–membrane assembly, mirror, laser source, projection screen, and recording device. (**B**) Frontal view of the projection screen and the video-capture setup, showing the projected laser pattern and the recording device positioned in front of the screen.

The audio files generated were played back using a custom-built device designed specifically for the experiment (Figure 2). This device consists of a tube with a Bluetooth-controlled speaker mounted at one end and a membrane with a circular mirror attached at the opposite end. After installation, the membrane was measured to have a surface tension of 152 N/m, ensuring the appropriate vibrational behavior required for the experiment. The method used to determine the membrane tension is as follows: A laser device positioned in front of the mirror projects a beam onto the mirror, which in turn reflects it onto a matte black screen. The vibration induced by the sonified EEG signal causes deviations in the laser’s position, generating dynamic projected patterns. 

The scene was captured using an iPhone 15 Pro high-resolution camera, resulting in a .MOV video file of approximately five minutes in duration (Alzheimer’s: File S3, Controls: File S4), matching the length of the generated audio for each group.

A: Side view of the device. B: Front view of the device. 

A Soundcore Mini 3 speaker (Anker Innovations Co., Ltd., Changsha, Hunan, China) was used, to which a plastic tube measuring 5 cm in depth and 8 cm in diameter was attached. A membrane was tightly placed over the opening of this tube, and at the center of the membrane, a self-adhesive circular mirror with a diameter of 2.5 cm was affixed. This part of the device was positioned at a 30-degree angle facing a black screen measuring 60 cm in height and 40 cm in width. In front of the mirror, a red 650 nm Ctricalver laser was placed, aimed directly at the mirror.

To estimate the surface tension of the membrane after installation, an acoustic method was employed. In this procedure, the membrane was subjected to acoustic excitation, and its fundamental resonant frequency was measured. The resonance frequency, corresponding to the first vibrational mode of the circular membrane, was then used to calculate the surface tension based on the physical properties of the system, as described below.

Surface density (*σ*) was determined gravimetrically as

σ=m/A

where m is the membrane mass and *A* = *π* a 2 is its area (with a being the radius). For *a* = 0.04 m and m = 2 g.

$$A\;=\;\pi {\left(0.04\right)}^{2}=5.03\times 10^{-3}\;m^{2},\sigma =\frac{0.002\;kg}{5.03\times 10^{-3}\;m^{2}}=0.398\;kg/m^{2}.$$

Membrane tension *T* was then calculated from the fundamental-mode resonance frequency *f*_01_ using the expression for a circular membrane (mode (0, 1)):

$$f_{01}=\frac{1}{2\pi }\frac{c_{01}}{a}\sqrt{\frac{T}{\sigma }}$$

where $c_{01}$ = 2.4048 is the first zero of the Bessel function $J_{0}$. Solving for *T* gives

$$T=\sigma {\left(\frac{2\pi af_{01}}{c_{01}}\right)}^{2}$$

Substituting *f*_01_ = 187 Hz yields

$$T=0.398\;kg/m^{2}\;{\left(\frac{2\pi \times 0.04\times 187\;Hz}{2.4048}\right)}^{2}\approx 152\;N/m.$$

#### 2.2.4. Computational Pipeline for Video Frame Preprocessing and Visual Pattern Isolation

In order to quantitatively analyze the visual dynamics of the laser projections, each video was subjected to a structured computational pipeline. This process involved the extraction of individual frames from the video recordings, followed by systematic binarization and enhancement procedures aimed at isolating the relevant visual patterns. The goal of this pipeline was to reduce noise, emphasize the laser trajectories, and prepare the data for subsequent spatial and temporal analyses.

##### Frame Extraction

**Figure 3:**
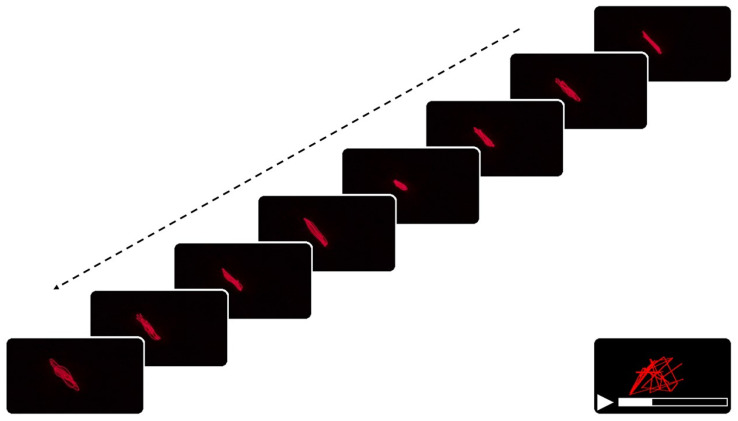
Frame extraction from laser projection video. Sequence of video frames extracted at 10 frames per second, illustrating the evolution of the laser projection over time. Each frame corresponds to a distinct time point in the recording, capturing the dynamic structure of the laser-induced pattern. The dotted arrow trajectory denotes the temporal progression of sequential frames, whereas the red intra-frame trace captures the laser’s oscillatory dynamics arising from mechanically induced vibration.The schematic at the bottom right represents the accumulation of these frames over time, emphasizing the temporal density and complexity of the projected trajectory.

To analyze the temporal evolution of the vibration patterns, the video files were processed and extracted into individual frames (Figure 3) using a custom Python 3.13 script with the OpenCV library (v. 4.11) [36], executed locally on a desktop computer. Each frame was saved as a .jpg (Joint Photographic Experts Group format) file at the original resolution and without additional compression. For the analysis of the laser projections generated from the sonified EEG signals, a video frame extraction process was implemented. Ten frames per second were extracted, which provided adequate temporal resolution to capture relevant dynamic changes without generating excessive redundancy or data saturation [37], and, therefore, not too much computational load. Each frame was saved as a .jpg file at the original resolution and without additional compression. To ensure temporal standardization and remove recording artifacts, a uniform 275 s segment was extracted from each video, starting from the onset of audible sonification. Initial trimming removed hardware initialization artifacts (first 12 s in AD video, first 0 s in control video due to faster equipment settling), and terminal segments were excluded to eliminate recording termination artifacts (last 15 s in AD video, last 4 s in control video). This procedure ensured that both groups contributed identical time (275 s) analysis windows, eliminating potential bias from differential recording durations while synchronizing the analyzed segments to the sonification onset.

##### Image Binarization and Structural Optimization for Quantitative Extraction

To reduce the complexity of the projected patterns and enable the extraction of quantitative metrics, each video frame was subjected to an adaptive binarization process. Specifically, a Gaussian adaptive thresholding method was applied, following the procedure described by Bradley and Roth [38]. The threshold *T (x*, *y)* for each pixel was calculated according to the formula:

T(x,y)=μ(x,y)−C

where *T (x*, *y)* is the locally adapted threshold for pixel position *(x*, *y)*, *μ (x*, *y)* is the mean intensity within a 31 × 31 pixel window centered at *(x*, *y)*, and *C* is a constant offset, set here to 2. This method ensures that thresholding remains sensitive to local illumination changes, which is particularly important given the natural variability in brightness and contrast inherent to the laser-based projection.

**Figure 4:**
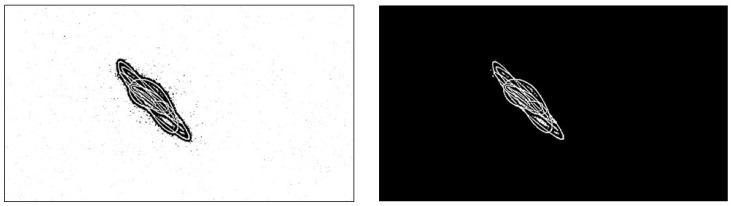
Adaptive binarization, morphological cleaning, and contour-based masking of a laser projection frame. Comparison between the initial adaptive binarization (left) and the result after the full structural optimization pipeline (right), applied to the same laser projection frame. In the left panel, numerous small black and white speckles can be observed scattered across the background, resulting from local thresholding. Although the adaptive method preserves spatial variations in illumination, it does not suppress spurious noise. The right panel shows the final cleaned and masked result, following morphological opening (3 × 3 elliptical structuring element) and the application of a contour-based envelope mask. This mask, computed from the largest connected contour in the inverted image, isolates the relevant trajectory while discarding peripheral and background artifacts, thereby improving the specificity of subsequent spatial analyses.

To improve the quality of the binarized images and facilitate the extraction of quantitative metrics, a morphological opening operation [39,40] was applied to each frame. This operation consists of erosion followed by dilation, using a 3 × 3 pixel elliptical structuring element. The purpose of this process is to eliminate small, isolated particles that do not belong to the main pattern, thus cleaning the binarized image (Figure 4). Morphological aperture is particularly effective in removing small objects from the foreground of an image while preserving the shape and size of larger objects [41]. This method has been widely used in image processing for denoising and improving the quality of binarized images [42].

Finally, to isolate the region corresponding strictly to the laser projection pattern, a contour-based envelope mask was computed. The binarized-cleaned image was first inverted, and then external contours were detected using the cv2.findContours function of OpenCV [36]. The largest contour by area was assumed to represent the primary vibrational structure and was used to generate a filled binary mask [43]. This mask was applied to the cleaned image, preserving only pixels within the main trajectory and excluding background or peripheral artifacts. This final filtering step ensured that all subsequent spatial analyses were restricted exclusively to the relevant projection region, significantly increasing the specificity and reliability of the extracted metrics.

#### 2.2.5. Quantitative Extraction of Spatial and Dynamical Features from Laser Projection Patterns

Each binarized and structurally optimized frame was subjected to a series of analyses aimed at quantifying spatial and temporal properties of the laser-projected patterns. The first metric calculated was the active area, defined as the number of white pixels (intensity 255) contained within the envelope mask, which represents the spatial extent of the vibrational pattern at a given instant. Formally, this measure is calculated as:

$$Active\;area=\sum \delta \left(Iᵢⱼ=255\right)$$

where *I*ᵢⱼ is the intensity value of the pixel at position (i, j), and *δ* is the Kronecker delta function, which is worth 1 if the condition is met and 0 otherwise.

In parallel, the spatial entropy of each frame was calculated using the classical Shannon entropy formula [44]:

$$H=-\sum_{\kappa }p\left(k\right)\;\log_{2}p\left(k\right)$$

where *p*(*k*) represents the probability that a pixel has an intensity *k*, which in this binary case corresponds to 0 or 255. This metric provides information about the degree of disorder or complexity of the image: high values indicate sparse or chaotic patterns, while low values reflect greater organization or concentration.

The fractal dimension of each pattern was also estimated using the classical box-counting method. For this purpose, the image was overlaid with a mesh of squares of decreasing size *ε*, the number *N(ε)* of cells containing part of the binarized pattern was counted, and the slope was fitted to a logarithmic space using the expression:

$$D=-\underset{\varepsilon \to 0}{lim}\frac{\log N\left(\varepsilon \right)}{\log\varepsilon }$$

The slope of the fit provides an estimate of the fractal dimension *D*, which quantifies the geometric complexity of the projected structure.

To characterize the dynamics of the pattern, the centroid of each frame was calculated by averaging the coordinates of the white pixels:

$$\left(x_{c},y_{c}\right)=\left(\frac{1}{N}\sum_{i}x_{i,}\frac{1}{N}\sum_{i}y_{i}\right)$$

where *N* is the total number of white pixels and (*x_i_*, *y_i_*) their respective coordinates. The centroid represents the center of mass of the vibration pattern at each instant.

Finally, by sequentially recording the centroids of all the frames, a temporal trajectory was reconstructed, reflecting the spatial evolution of the projected pattern over time. From this trajectory, absolute displacements (representation in the XY plane), accumulated displacements and displacements between consecutive frames were calculated, which made it possible to detect oscillations, directional changes or expansion and contraction patterns in the laser dynamic.

#### 2.2.6. Machine Learning Classification Analysis

To evaluate the separability of dynamic patterns derived from sonified averaged EEG signals between healthy subjects and Alzheimer’s disease patients, a supervised classification approach was implemented using a Random Forest model [45]. Random Forest is an ensemble machine learning algorithm that constructs multiple decision trees during training and combines their outputs, typically through majority voting, to improve predictive accuracy and reduce overfitting. Each tree is built from a random subset of the data and features, introducing diversity that enhances generalization and model stability [46]. The Random Forest algorithm was chosen for its interpretability, robustness to overfitting, and strong performance with nonlinear relationships and mixed-type features, characteristics particularly suitable for the multidimensional descriptors. Furthermore, its ensemble nature mitigates variance and bias issues common in small or moderately sized biomedical datasets [47,48].

##### Data Partitioning Strategy

The complete dataset of processed video frames was systematically divided using a holdout validation approach. Eighty percent of the images (6044 samples) were allocated for model training and internal validation, while the remaining twenty percent (1512 samples) were reserved as an independent test set for final performance evaluation. This strict separation protocol ensured that the test dataset remained completely unseen during the model training and hyperparameter optimization phases, thereby enabling an unbiased assessment of the model’s generalization capability.

##### Model Robustness Validation

To ensure robust model performance and prevent overfitting, multiple validation strategies were implemented:

Cross-validation procedure: A stratified 5-fold cross-validation was performed on the training dataset. This approach [49,50] involved partitioning the training data into five equal subsets, ensuring balanced representation of both classes (healthy controls and Alzheimer’s patients) in each fold. The model was iteratively trained on four folds and validated on the remaining fold, with this process repeated five times to obtain comprehensive performance estimates across different data partitions.

Noise injection protocol: During the feature extraction phase, Gaussian noise with a standard deviation of 5 intensity units (on a 0 to 255 pixel intensity scale) was systematically introduced [51,52] to the processed images. This controlled perturbation strategy was designed to simulate non-ideal acquisition or processing conditions, thereby testing the model’s robustness against minor variations in input data quality.

Overfitting assessment: Model generalization was evaluated by comparing cross-validation performance metrics with those obtained from the independent test set, ensuring no significant performance degradation that would indicate overfitting to the training data.

##### Classification Algorithm and Evaluation Metrics

The Random Forest algorithm was selected as the primary classification method due to its robustness against overfitting and ability to handle high-dimensional feature spaces. Model performance was assessed using standard binary classification metrics, including accuracy, precision, recall, and F1-score for both classes (healthy controls and Alzheimer’s patients). Table 1 shows the Random Forest parameters used.

##### Performance Analysis

The model’s discriminative capacity was further evaluated through Receiver Operating Characteristic (ROC) curve analysis, with the Area Under the Curve (AUC-ROC) calculated as a comprehensive measure of classification performance. A confusion matrix was generated to provide detailed insights into the model’s classification behavior for both classes.

#### 2.2.7. Statistical Analysis Framework

All performance metrics were calculated with confidence intervals where applicable, and the stratified cross-validation results were reported as mean ± standard deviation to indicate model stability across different data partitions. The final model evaluation was conducted exclusively on the reserved independent test set to provide an unbiased estimate of real-world performance.

## 3. Results

In order to evaluate the discriminative ability of the trained Random Forest model to distinguish between dynamic patterns derived from the sonification of average EEG signals of healthy subjects and patients with Alzheimer’s disease, a comprehensive evaluation was performed using multiple performance metrics and validation techniques.

### 3.1. Stratified Cross-Validation

The 5-fold stratified cross-validation performed on the training set showed remarkable consistency in model performance. The results are presented in Table 2.

### 3.2. Evaluation in Independent Test Set

The final evaluation of the model was performed on a completely independent test set, consisting of 1512 samples (20% of the total dataset). This set was not used during any phase of training or hyperparameter fitting, ensuring an unbiased evaluation of the model’s generalizability.

The confusion matrix obtained in the final evaluation is presented in Table 3, showing the distribution of correct and incorrect predictions for both classes.

### 3.3. Performance Metrics

To comprehensively assess the classification performance, several standard metrics were computed from the confusion matrix. Accuracy measures the overall proportion of correctly classified samples, while Recall quantifies the ability to correctly identify positive cases. Specificity represents the proportion of correctly identified negative cases, and the F1-Score provides a harmonic balance between Precision and Recall, offering a robust measure of performance for imbalanced datasets.

Additionally, the ROC curve and the Area Under the Curve (AUC-ROC) were used to evaluate the model’s discriminative capability across all possible classification thresholds. The ROC curve plots the True Positive Rate against the False Positive Rate, and the AUC-ROC summarizes this relationship into a single value, where values close to 1.0 indicate excellent discrimination between classes [53,54,55].

Detailed performance metrics calculated from the confusion matrix are presented in Table 4. These metrics provide a comprehensive assessment of the discriminative ability of the model for both classes.

### 3.4. ROC Curve Analysis

**Figure 5:**
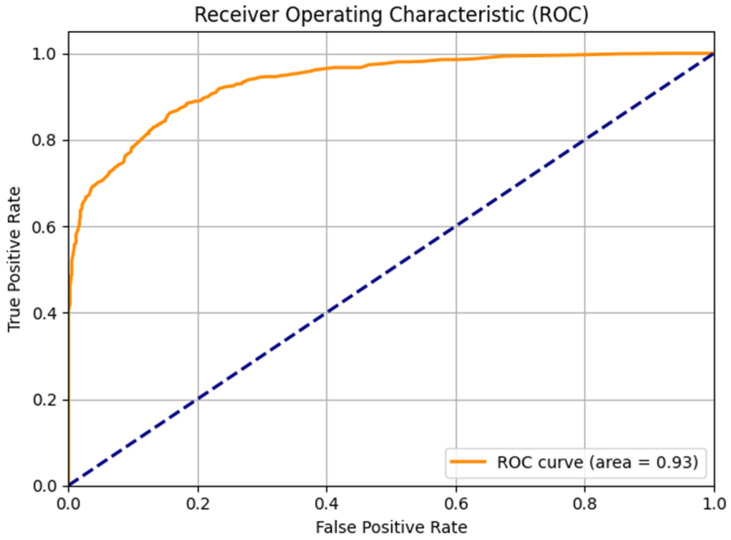
ROC curve of the Random Forest model for classification of dynamic patterns derived from sonified EEG. Note: The AUC-ROC of 0.93 indicates an excellent discriminatory ability between control subjects and Alzheimer’s patients.

The ROC curve analysis (Figure 5) revealed a good discriminative ability of the model. The ROC curve obtained is characterized by a rapid initial rise towards the upper left corner of the graph, indicating a high rate of true positives with a low rate of false positives across different classification thresholds.

An AUC-ROC = 0.93 was obtained, which represents a good performance according to standard interpretation criteria (AUC > 0.9 indicates excellent discrimination). This value confirms the high ability of the model to distinguish between the average visual dynamic patterns derived from the sonified EEG signals of healthy subjects and patients with AD.

#### Model Robustness Evaluation

Implementation of robustness techniques during training, including controlled injection of Gaussian noise (σ = 5 intensity units) and stratified cross-validation, demonstrated the ability of the model to maintain stable performance in the face of minor variations in the input data. Comparison between the cross-validation results (accuracy = 0.8400 ± 0.0120) and the evaluation on the independent test set (accuracy = 0.85) suggests an absence of overfitting, with a slight improvement in final performance that can be attributed to natural optimization of the training process.

## 4. Discussion

The results obtained demonstrate that the proposed methodological approach, based on the sonification of average EEG signals and their subsequent transformation into visual dynamic patterns, constitutes a viable and effective strategy for automated discrimination between control subjects and patients with AD. The trained Random Forest model achieved consistently high performance metrics, with an adequate balance between sensitivity and specificity for both classes, suggesting its potential clinical applicability as a diagnostic support tool.

The stability observed in the different validation schemes, together with the excellent AUC value, supports the robustness and reliability of the presented findings, providing evidence of the effectiveness of the proposed method for the classification of neurophysiological patterns in the context of AD. In the context of EEG-based Alzheimer’s disease classification, reported accuracies in the literature span a wide range (e.g., from approximately 77% up to 96.9%) [56,57,58]. Therefore, the present model’s performance (accuracy = 0.85, AUC = 0.93) is consistent with that spectrum of results, lying in the mid-to-high zone, which suggests an adequate and competitive discriminative capability for this domain.

In this study, we employed a pure direct mapping sonification approach where EEG signals were directly translated into sound, with amplitudes of the EEG mapped linearly to sound pressure levels. This method preserves the original temporal structure of the brain activity without introducing complex transformations. In contrast, some previous studies involving EEG sonification, such as those by Inoue [29] and Wu et al. [59], used extended mapping approaches. These included additional processes such as emotion-based musification or logarithmic scaling to make the sound more perceptible to human hearing. Similarly, other works like Hermann & Meinicke [25] employed spectral mapping sonification, which also involves more complex signal transformations compared to our direct approach.

Our methodology stands out for its strict adherence to direct mapping, making it a simpler and more faithful representation of the original EEG data compared to these other approaches. By not introducing emotional cues, distance codings, or logarithmic alterations, we ensure that the sound output retains a more accurate and undistorted representation of the brain’s electrical activity, potentially offering clearer insights into neural dynamics.

The proposed approach theoretically offers fundamental advantages that transcend the inherent limitations of direct EEG analysis. Whereas conventional analysis relies on the extraction of statistical and spectral features from two-dimensional electrical signals [22], presenting critical limitations in characterizing the inherent complexity of brain signals [23], our method converts these signals into three-dimensional spatiotemporal patterns that can reveal dynamics hidden in the purely electrical domain. The physical conversion of sound into vibrational patterns using the laser membrane system introduces a completely new dimension to neuropsychophysiological analysis. This process transforms EEG signals into dynamic spatial patterns that can be quantified by geometric and fractal metrics impossible to obtain directly from digital EEG. The analysis of active area, spatial entropy, fractal dimension and centroid dynamics provides complementary descriptors that capture aspects of neural organization that remain hidden in traditional spectral analysis.

A limitation of conventional EEG analysis is its tendency to fragment the signal into discrete temporal windows for spectral analysis or feature extraction. This approach, while computationally efficient, can miss crucial information about the global dynamics and long-range temporal correlations that characterize brain activity [21].

Our direct sonification method preserves the full temporal continuity of the averaged EEG signal, keeping the temporal relationships between different time scales intact. The resulting vibrational patterns represent a continuous temporal signature that captures not only the amplitude and frequency of brain oscillations but also their nonlinear interactions and long-range phase modulations. These complex temporal features may be particularly relevant in AD, where neuronal desynchronization and loss of temporal coherence are pathognomonic findings [60,61,62].

Our results demonstrate that the proposed methodological approach constitutes a viable and effective strategy for the automated discrimination between control subjects and patients with AD. The model’s discriminative capacity suggests that the dynamic patterns derived from sonification capture neurophysiologically relevant information, likely extending beyond conventional EEG measures due to the method’s ability to preserve complex temporal relationships and transform them into quantifiable spatial descriptors. The transformation of EEG signals into dynamic spatial patterns through our system introduces an entirely new dimension to neuropsychophysiological analysis. This process converts EEG signals into dynamic spatial configurations that can be quantified using geometric and fractal metrics not directly obtainable from digital EEG. The analysis of active area, spatial entropy, fractal dimension, and centroid dynamics provides complementary descriptors that capture aspects of neural organization that remain hidden in traditional spectral analysis. Changes in the fractal dimension of the projected patterns may reflect alterations in the complexity of synchronized neural activity. Under normal conditions, brain activity exhibits fractal properties that mirror the hierarchical organization of neuronal networks [63,64]. A reduction in fractal complexity in patterns derived from patients with AD may indicate a pathological simplification of these dynamics, consistent with the synaptic loss and network disorganization characteristic of the disorder.

The attainment of high, albeit improvable, performance metrics, with an appropriate balance between sensitivity and specificity for both classes, suggests the potential clinical applicability of the approach as a diagnostic support tool. The non-invasive nature of EEG, combined with the proposed automated processing, could facilitate the development of accessible and cost-effective screening systems for the early detection of AD.

A major limitation of the present study lies in the use of group-averaged EEG rather than individual-level EEG. This methodological choice was intentional, as the main objective of the system at this stage was to test the feasibility of translating neural electrical activity into stable physical patterns under controlled conditions. Averaging improved the signal-to-noise ratio and reduced individual variability that could obscure the detection of systematic differences at the group level. However, this approach inherently eliminates inter-subject variability and prevents the evaluation of diagnostic performance at the individual level. Therefore, the current results should be interpreted as a demonstration of the feasibility and discrimination of patterns at the group level, rather than as individual biomarkers.

A second phase of this research, currently underway, addresses this limitation by applying the same analytical process to individual EEG recordings and independent test cohorts. This will allow for an adequate estimation of inter-subject variability, model generalization, and potential clinical applicability.

Another limitation is related to the model performance and its interpretation. The Random Forest classifier achieved an AUC of 0.93, indicating strong discriminative capacity between the dynamic patterns of AD and control groups. However, this result should be interpreted as a methodological validation rather than an optimized benchmark. No comparative evaluation with alternative classifiers (e.g., SVM, CNN) or feature sets was conducted, as the primary aim of the study was to establish the feasibility of the biomimetic transduction pipeline rather than to maximize classification accuracy. Future work will extend this analysis by incorporating additional classifiers and cross-modal feature representations to assess the robustness and scalability of the model. Additionally, future work will include systematic comparisons with conventional EEG spectral features (power spectral density in standard frequency bands) and audio-derived features (MFCCs, spectral centroid) to establish the relative contribution of the physical transduction process.

Similarly, bootstrap confidence intervals for ROC curves, precision–recall analysis, and feature importance assessments (e.g., permutation importance, SHAP values) will be implemented in the individual-level validation phase to provide robust statistical inference about classification performance and feature contributions. While the current proof-of-concept demonstrates discriminative capacity at the group level, these advanced statistical evaluations are essential for establishing reliability and interpretability in individual-level diagnostic applications.

The visual descriptors used—entropy and fractal dimension—were selected for their capacity to capture signal complexity and spatial–temporal irregularity, features known to reflect neural disorganization and loss of complexity in Alzheimer’s disease [33,65,66,67,68]. Reductions in EEG entropy and fractal dimension have been consistently associated with diminished functional connectivity and impaired neural synchronization in AD. Thus, while the present approach does not directly model underlying neurophysiological mechanisms, the extracted metrics align with established electrophysiological alterations, providing an interpretable link between the visual dynamics of the projected patterns and known cortical dysfunction in Alzheimer’s pathology.

On the other hand, the dataset used consisted of 65 EEG recordings obtained from a single open-source repository (OpenNeuro, dataset ds004504). While the use of an openly available dataset ensures methodological transparency and reproducibility, it restricts population diversity and therefore limits the generalizability of the findings.

Additionally, although more than 7000 video frames were analyzed, these frames originate from a small number of EEG recordings and cannot be considered independent samples. The frame decomposition was implemented to characterize the temporal evolution of the vibration–laser patterns derived from these group-level signals, not to artificially increase the number of effective samples. Consequently, the present results should be interpreted as reflecting group-level differences rather than individual-level variability.

This approach is consistent with exploratory or proof-of-concept studies in computational neuroscience, where group-averaged signals are used to test the feasibility of novel analytical frameworks before progressing to subject-level validation [69,70,71]. Nonetheless, we acknowledge that a larger and more heterogeneous dataset, including independent recordings and individual-level analyses, will be essential in future work to assess the reproducibility and clinical generalizability of the proposed framework.

The system employed, although innovative, requires precise calibration and strict control of environmental conditions, which may limit its implementation. Future developments should aim at the miniaturization and standardization of the system to facilitate its adoption in both research and clinical contexts, should the latter prove of interest. Moreover, investigating different membrane configurations and laser projection parameters could optimize the sensitivity of the method for detecting distinct types of neurophysiological alterations.

The integration of the proposed method with other AD biomarkers represents a promising direction for future studies. Blood-based biomarkers of tau and amyloid-β, combined with vibrational EEG pattern analysis, could provide a more robust multimodal approach for characterizing disease progression. Furthermore, the combination with advanced neuroimaging techniques such as amyloid- or tau-PET could allow correlation of dynamic patterns with specific pathological burden in different brain regions. This would contribute to a deeper understanding of the neurobiological mechanisms underlying potential differences in vibrational patterns.

The method developed could have significant implications for the monitoring of therapeutic interventions in AD. The ability to detect subtle changes in dynamic brain patterns may enable the objective evaluation of pharmacological treatments or non-invasive brain stimulation approaches.

Finally, vibrational patterns could serve as biomarkers of treatment response, allowing therapeutic adjustments before overt cognitive changes become apparent. This conceptual bridge, which translates internal neural dynamics into measurable external vibration patterns, represents a novel contribution, as no previous study, to our knowledge, has physically modeled the brain’s electrical activity in real space-time projections. This biomimetic analogy goes beyond visualization and extends to a real mechanical correspondence between the dynamics of brain signals and the formation of physical patterns.

## 5. Conclusions

This work shows that direct EEG sonification combined with vibration–laser projection yields dynamic visual patterns containing measurable features that differentiate Alzheimer’s disease patients from healthy controls. The extracted descriptors, together with machine learning classification, demonstrated robust discriminative capacity. These findings highlight the potential of this novel methodology as a non-invasive tool for supporting diagnosis and monitoring. Moreover, by translating neural electrical activity into physical spatiotemporal patterns, the method draws on a biomimetic principle—converting internal signals into perceptible representations—thus reinforcing its conceptual and interdisciplinary relevance. Future studies should validate its applicability at the individual level and in larger cohorts.

Beyond its diagnostic potential, the present approach also illustrates how biomimetic principles can inspire new analytical frameworks in neuroscience and biomedical engineering. By mimicking how biological systems convert electrical activity into sensory representations, this method provides a functional link between neural signal processing and the generation of physical patterns. Such translation not only facilitates human interpretation of complex brain dynamics but also opens avenues for developing hybrid analytical systems that integrate biological signal processing with physical media for visualization and exploratory modeling.

## Figures and Tables

**Table 1:** Random Forest parameters.

Category	Parameter	Value
Random Forest Classifier	n_estimators	100
	random_state	42
	max_depth	None
	criterion	‘gini’
	min_samples_split	2
	min_samples_leaf	1
	bootstrap	True
	max_features	‘sqrt’

**Table 2:** 5-Fold Stratified Cross-Validation Results.

Metrics	Mean	Standard Deviation	Confidence Interval (95%)
Accuracy	0.8400	0.0120	0.8280–0.8520

Note. The results show the average of five stratified cross-validation rounds while keeping the percentage of classes in each division.

**Table 3:** Confusion Matrix of the Random Forest Model in Test Set.

Real Class	Predicted Class		Total
	Control	Alzheimer	
**Control**	**645**	108	753
**Alzheimer’s**	122	**637**	759
**Total**	767	745	1512

Note: The values in bold represent the correct classifications.

**Table 4:** Model Performance Metrics by Class.

Class	Accuracy	Recall	F1-Score	Specificity
Control	0.84	0.86	0.85	0.84
Alzheimer’s	0.86	0.84	0.85	0.86
Average	0.85	0.85	0.85	0.85

Note: All metrics show balanced values across classes, indicating no bias toward a specific class. Overall Accuracy = 0.85.

## Data Availability

The data that support the findings of this study are publicly available on Zenodo: Dataset DOI: 10.5281/zenodo.17555481.

## Supplementary Materials

The following supporting information can be downloaded at https://doi.org/10.5281/zenodo.17639457.

## Note

This work is part of Rubén Pérez-Elvira’s PhD thesis in medicine and surgery.

## Abbreviations

The following abbreviations are used in this manuscript:

AD	Alzheimer’s Disease
EEG	Electroencephalogram
PET	Positron Emission Tomography
